# Measuring Counterintuitiveness in Supernatural Agent Dream Imagery

**DOI:** 10.3389/fpsyg.2019.01728

**Published:** 2019-08-09

**Authors:** Andreas Nordin, Pär Bjälkebring

**Affiliations:** ^1^Department of Cultural Sciences, University of Gothenburg, Gothenburg, Sweden; ^2^Department of Psychology, University of Gothenburg, Gothenburg, Sweden

**Keywords:** dreaming, cognition, counterintuition, supernatural agent concept, religion, CI scheme

## Abstract

The present article tests counterintuitiveness theory and methodology in relation to religious dream imagery using data on religious dream content. The endeavor adopts a “fractionated” or “piecemeal” approach where supernatural agent (SA) cognition is held to be a pivotal building block of purportedly religious dreaming. Such supernaturalistic conceptualizations manifest in a cognitive environment of dream simulation processes, threat detection, and violation of basic conceptual categorization characterized by counterintuitiveness. By addressing SA cognitions as constituents of allegedly religious dream imagery, additional theorizing and supporting data are presented in a growing body of research in the cognitive science of religion (e.g., Barrett et al., 2009;Hornbeck and Barrett, 2013;Barrett, 2017) and on religious dreaming (McNamara and Bulkeley, 2015;McNamara, 2016). The aim of the article is partly to map and align contemporary theorizing regarding counterintuitiveness and CI schemes with empirical qualification of the prosaic hypothesis about the predominance of supernaturalism in allegedly religious dreaming. This is done by (1) exploring the crucial topic of the pervasiveness of cognitive counterintuitiveness; (2) testing Barrett’s counterintuitiveness coding and quantifying scheme (CI scheme) for counterintuitiveness in the context of religious dreaming by assessing intercoder reliability; and (3) exploring the prevalence and base rate frequency of counterintuitiveness in dream reports. This undertaking aims to contribute to the methodology and understanding of religious dream cognition, as well as to establish the cross-cultural base rates of counterintuitiveness in dreams for future research.

## Introduction

Supernatural agent concepts consistently appear in dreams in association with diminished agency in the dreamer (who thus loses individual agency and ego), which may facilitate supernatural agent cognitions related to religious beliefs. The anthropological and religious research literature tends to demonstrate that dreaming, dream experience and narratives found in traditional societies connect to other religious ideas and practices (e.g., Tylor, 1871; Lincoln, 1935; Tedlock, 1987; Jedrej and Shaw, 1992; Doniger and Bulkeley, 1993; Mageo, 2003; Lohmann, 2003a,b; Bulkeley, 2007, 2008; Laughlin, 2011). Based on analysis of lived and practiced folk-religion from various parts of the world, a notion has developed among scholars characterizing *dreaming as the primary source of religion*. Such a single “magic bullet” theory is ambiguous, however. A strong case can be made for the notion that supernaturalistic cognition prevails in dreaming processes connected to cultural environments rich in other religious representations. The aim here is to map a particular structure of cognitive content using Barrett’s counterintuitiveness coding and quantifying scheme (CI scheme) in allegedly religious dreaming by focusing on supernatural agent imagery and concepts. Are these types of items in subjective dream reports a similar kind of catchy and peculiar content as has been demonstrated from the cultural transmission of other supernaturalistic notions and religious ideas generally? If it can be shown that allegedly religious dream imagery involves similar counterintuitive processing, this would help further explain why dreams cross-culturally tend to have a certain salience, and why these types of experiences and imagery are rendered special value and usefulness in cultural and religious institutions. From the perspective of the broader naturalistic research program of cognitive (and evolutionary) science of religion, many of the kinds of characteristics that occur in these dreams are *supernatural agent* (SA) concepts. The use of “superhuman” or “supernatural” agents (e.g., ancestors, gods, ghosts, sprits, souls, demons, saints, and even religious experts) is an indication that these notions hold at least an important, and perhaps a constitutive, position in religious beliefs and ritual action (c.f., McCauley and Lawson, 2002). Further, religious dreaming draws upon the same neurocognitive mechanisms as do ordinary dreaming and cognitive processing, as well as the cultural transmission biases that amplify and contextualize the spreading and preservation of representations about dreaming. It has been noted that, with some exceptions (e.g., Bulkeley, 2007; Nordin, 2011; McNamara and Bulkeley, 2015; McNamara, 2016), dreaming has not garnered much attention in the cognitive science of religion and related fields (c.f., Bulkeley, 2004, p. 22; Taves, 2008). While anthropological literature points to the cross-cultural pervasiveness of supernaturalism in dreams, and the cognitive science of religion has paid a lot of attention to the cognitive and or functional peculiarities of supernaturalism, there are very little cross-cultural data and research on the cognition of supernatural dreaming. Religious and supernatural agent dream imagery tends to be ritualized, and is awarded special (sacred) value and cultural institutionalization related to interpretive rationalization and *modus operandi*. But why should dreaming produce supernatural agent imagery? It has been suggested that this sort of imagery and related emotional activation are generated from evolved threat simulation processes during sleep (Bulkeley, 2007; Nordin, 2011). Such a system is perhaps part a grander evolved system for agent-related threat detection (Nordin, 2011) that has been described as the hypersensitive agent detection device or HADD (Barrett, 2004a,b; c.f., Guthrie, 1993) and is tied to theory of mind attribution. Such a model suggests that HADD and threat simulation make use of an increased inclination to simulate and detect agency in situations of urgency and uncertainty. Dreams and nightmares with salient content, strong negative emotions, and existential anxiety create urgent concern. Perhaps the sensed urgency in dreams would contribute to supernatural beliefs, since these concepts are particularly salient and inferentially rich (strategic knowledge and morality); and are usually already available in the believer’s explanatory repertoire. And consequently, the intentions of extraordinary agents with extraordinary properties are likely to be believed to be the cause or content of the dream (Nordin, 2011). This may be the case because, according to HADD, salient dreams may be conceived as traces or communicative signs from other agents (Barrett, 2004a,b). However, supernatural agent dream imagery is often manifestly extraordinary in some way, and it is consequently not obvious that such imagery connects to inferences that imply a maximal potential for cognitive relevance.

On the other hand, why does the dream cognition start to represent extraordinary or counterintuitive properties? Neurocognitive theories further suggest that the combination of dream simulation, theory of mind attribution, and high dopaminergic/low serotonergic activation generates special (religious) value hierarchies (McNamara and Bulkeley, 2015; McNamara, 2016) in REM sleep at the same time as a significant deactivation of the prefrontal cortex occurs. Such states would seem to deplete the attributions of an agentic self-model being the cause of the dream events while at the same time exaggerating the salience of a sense of agency attributed to supernatural characters in the dream (McNamara and Bulkeley, 2015; McNamara, 2016).

Still, questions remain about why prediction or simulation would construct the dream items with extraordinary or counterintuitive properties. Whether when dreaming or awake, how and why are supernatural agent concepts and imagery given their peculiar cognitive properties? In the cognitive (and evolutionary) science of religion, SA concepts and cognition have gained a lot of attention and been theorized and researched according to the phenomena of counterintuitive processing and minimal counterintuitiveness (MCI) theory (e.g., Boyer, 2001; Barrett, 2004a,b). Minimally counterintuitive concepts violate certain intuitive assumptions about properties of categories such as agents, objects, and artifacts, rendering such concepts more salient, memorable, and inferentially rich, and consequently more likely to successfully achieve cultural transmission. Entities such as SAs can have either an overly excessive counterintuitive effect, making it difficult to remember characteristics and hence retrieve and communicate the concepts, or an overly weak counterintuitive effect, weakening the impression and hampering memorability. There is ample evidence of the prevalence of SAs in dreams from around the world and their constitutive relation to other “religious traits.” Such supernaturalism suggests the operation of (minimally) counterintuitive processing. However, what if all dreams (even those not tied to other “religious traits”) actually contain counterintuitive imagery processing as a subcategory of a general dream-symbolization (c.f., Brereton, 2000)? Might so-called “religious dreams” instead reflect a general tendency of cultural schemata and institutionalization to adopt certain recalled fragments from a broader pool of counterintuitive dream “stuff”? MCI theory seems to imply that not all catchy and attention-grabbing ideas are counterintuitive, but some are, notably those connected with religious traits that contain supernatural agent imagery.

These observations raise general concerns about (1) why dreaming should produce supernatural agent imagery, and specifically (2) why supernatural agent dream imagery presents itself as counterintuitive; and further (3) empirically, to what extent is religious and supernatural agent dream imagery actually counterintuitive? The purpose of this article is partly theoretical, dealing with the issue in (1) + (2), and in doing so offering empirical support for topic (3) by adopting and testing Barrett’s methodology and coding scheme for counterintuitivity and its intercoder reliability. This is accomplished by examining reports from Hindu Nepalese respondents on the allegedly counterintuitive content in “religious dreaming.” In particular, a report is offered on the prevalence, modalities, and types of counterintuitive dream imagery described by Hindu Nepalese respondents. This article presents interrelated strands of research related to dreaming, religion, and cognition, such as the neurocognition of dreams, anthropological research on supernatural dreaming, and models of supernatural agent cognition from the field of the cognitive science of religion. The discussion concludes by adopting and testing Barrett’s coding scheme for counterintuitivity to explain and provide empirical support for the prevalence and modalities of counterintuitive dream content.

### Dreaming in Anthropological and Religious Research

Anthropological analysis has suggested the notion that dreaming is the primary source of religion. This is recurrently observed in religious ideas and practices in traditional cultures (e.g., Tylor, 1871; Tedlock, 1987) and further implies that dreams are useful tools for spreading and transmitting religious ideas (Knafo and Glick, 2000). The early and classical accounts in Tylor suggested that dreaming was the experiential source of religious beliefs based on the observation of a nearly universal belief that dreams are experiences of, and communication with, “real” souls, spirits, ancestors, gods, and other supernatural entities (Tylor, 1871). These approaches suggest that it is likely that early human populations (and probably human populations ever since) deployed dreams as *evidence* for supernatural entities and realms, and such convictions drew upon the *vivid emotional experience*, sense of “realness,” and *involuntary encounter* with others. Such evidence is cross-culturally manifested in “visitations” – dreams that combine an intense sense of realness with strongly apprehensive or non-apprehensive experience.

Anthropologists have contributed to the understanding of how cultural environments tend to emphasize different dream models concerned with themes of: “nonsense”/bizarreness; discernment; semiotic messages; generative precognition; “soul travel”; and visitation in dreams (Lohmann, 2007, pp. 41–44). Furthermore, the importance of the pragmatic and communicative context of the dream and the dreamer has been emphasized (Tedlock, 1987). Consequently, different discursive or explicit cultural models exist, and according to Kilborne, seven (overlapping) dream classification systems are common, based on how dreams are perceived, used, and connected to social dynamics and pragmatic concerns (Tedlock, 1987, p. 174). For example, dreams can have divinatory, political, religious, artistic, formal, therapeutic, psychodynamic, or expressive functions. These different uses of dreams illustrate the values attached to supernatural agent concepts and have been demonstrated in various ethnographic descriptions (e.g., Jedrej and Shaw, 1992; Peluso, 2004; Renne, 2004). However, these approaches and descriptions are slightly weak theoretically, and provide no information about the structure, content, and distribution of cognitive cultural schemas and scripts associated with religious dreaming.

The ethnographic literature points to the widespread cultural value attached to supernatural agent concepts and dreams (Jedrej and Shaw, 1992; Littlewood, 2004; Peluso, 2004; Renne, 2004) and their importance in all the world’s religious traditions (Doniger and Bulkeley, 1993; Bulkeley, 2007, 2008, 2009). Studies of Taliban Jihadists (Edgar, 2004), independent churches in Nigeria (Renne, 2004), Trinidadian Baptists (Littlewood, 2004), and Jewish nationalism (Knafo and Glick, 2000) have noted the importance of religious dreams and the tendency to institutionalization. “The idea of certain status and value relates to the culturally widespread notion that dreams serve as anchors of belief conviction by offering direct experiential verification of religious entities and a spirit realm” (Bulkeley, 2008). Noteworthy results from cross-cultural ethnographic surveys of dreams highlight the importance of cultural traits that relate dreams to religious systems (D’Andrade, 1961). There is widespread use of dreams to contact or gain control of supernatural (agents) powers, and there are prevalent beliefs about the soul wandering during sleep and meeting other souls. D’Andrade’s study shows that anxiety about being alone, demands of self-reliance, and isolation give rise to powerful preoccupations with supernatural dreams (D’Andrade, 1961. pp. 320, 328). It is often held that the dreamer’s soul visits the spirit world and communes with gods and spirits (e.g., Lohmann, 2003a,b). Furthermore, in various cultures and religious traditions, dreams and nightmares are employed by shamans, healers, prophets, and oracles in local disciplines and rituals such as cults of pilgrimage, initiations, and conversion ordeals (Morinis, 1982; Bulkeley, 2007; Nordin, 2011). Nightmares are often held to be warnings from spirits, ancestors, God or the gods, or demons. Ethnographic accounts from various traditions in which ancestor worship is common practice also note that dead ancestors appear in frightening and memorable dreams to reprimand the dreamer for failing to perform commemorative rituals (e.g., Trompf, 1990; Jedrej and Shaw, 1992; Boyer, 2001).

### Supernaturalism, Supernatural Agent Concepts, and the Category of “Religion”

From a cross-cultural and cognitive perspective, religious notions and supernaturalist imagery share recurrent features. Within the research program cognitive science of religion, minimal counterintuitiveness (MCI) theory was developed to explain the cognitive features and cultural transmission success of supernaturalistic representations. The prevalence of counterintuitiveness in supernaturalist and religious contexts has been amply demonstrated, while at the same time the general theory is under constant revision, through confrontation with empirical and ethnographical testing and debate.

A key question in this article is: to what extent do religious dream imagery and narratives contain counterintuitive properties of this sort, given the general predominance of supernaturalism in religious dreaming? This study starts from the methodological assumptions that the category of “religion” encompasses a broad range of phenomena that must be “fractionated” into constitutive parts. “Religion” is, anthropologically speaking, not a unitary phenomenon (e.g., Bloch, 2008; Boyer, 2013; Sperber, 2017) but a cluster of more basic and recurrent traits and cultural expressions that do not co-occur in every social environment. These elements – their recurrence and relationships – can then be addressed separately, making them more amenable to scientific investigation (e.g., Atran, 2002; Boyer, 2005; Sørensen, 2005). This is a kind of “piecemeal” (Barrett, 2007) or “building-blocks” (Taves, 2011) approach to the research area that explores targeted exemplars of cultural expressions that are seemingly related in cause or effect from the perspective of prominent explanatory models such as evolutionary cognitive and psychological accounts (regardless of any purported cultural or ideological systemization of the items).

### Sleep States and Some Neurocognitive Correlates to (Religious) Dream Production

Dreaming is a universal human experience, but despite the mundane belief that dream content is mostly unintelligible and bizarre, research shows that it is structured in relation to activities, thought, and feelings from waking life (e.g., Hall and Van de Castle, 1966; Revonsuo, 2000a,b; Domhoff, 2003; Bulkeley, 2007). By dreams can be meant mental/emotional imagery and representations that occur during sleep. A dream can be defined as a subjective experience that occurs during sleep and consists of a temporal progression of images (c.f., Revonsuo, 2000a, p. 878). The literature points to various theories regarding the functions of dreaming (for an overview see Bulkeley, 1997; Revonsuo, 2000a). Neurocognitive models (e.g., Foulkes, 1985; Hobson, 1994; Solms, 1997) usually stress the randomness of dream imagery and the epiphenomenal and non-functional nature of dream *content* (c.f., Revonsuo, 2000a, p. 880). By contrast, psychological theories tend to regard dreams as a way for the individual to cope psychologically with the conditions of his/her current waking life and as promoting well-being (e.g., Jung, 1933). Dreaming is thus seen as serving as a means of solving intellectual or emotional problems and enabling use to adapt in order to cope with problems faced in waking life (e.g., Breger, 1967). Other research suggests that dreaming does not help provide solutions to problems in daily life (Blagrove, 1992), though this may not apply to religious folk models of dream function (and the alleged placebo effects of religious dreams).

Although most dreaming seems to occur during REM (rapid eye movement) sleep, the mind-brain system is active during the entire sleep cycle, indicating that we dream more or less all night long (Bulkeley, 2007), even during NREM (non-REM) sleep states (e.g., McNamara and Bulkeley, 2015).

Research by Hobson et al. (2000) demonstrates the complex and distinct quantitative variance between REM and NREM processing, and between REM and mentation during waking states. Some of these differences are manifested in the fact that affective and intensely emotional experiences are generally much stronger in REM than in NREM states (Hobson et al., 2000; Scarpelli et al., 2019). Additionally, Hobson et al. (2000) summarize physiological characteristics that differ between REM, NREM, and waking states: (1) during NREM nightmares, autonomic activation is higher than during REM night terrors; (2) despite the predominance of anxiety in the emotions of dreaming during REM sleep, the locus coeruleus region is inactive, even though that region is active during anxiety responses in the waking state connected with noradrenergic output; (3) the dream anxiety typical of REM sleep is likely to be underpinned by cholinergic activation in the limbic system, which may not be as prominently engaged in anxiety during waking states; (4) REM and NREM sleep states also discharge differing stimulus responses and cognitive processes, some of which will be further elaborated upon below. Alternatively, Kirov et al. (2012) consider the differences between the waking state, NREM sleep, and REM sleep to be about variance in connectivity, neuroelectric signaling, mentality, and neurochemistry. Further, Kirov et al. note that, under conditions of REM sleep, emotions emerge outside of a goal-directed behavioral context and with a lack of external input, and their conceptual and motivational schemata are opaque and vague during these states (Kirov et al., 2012).

The research literature on the neurobiological mechanisms of REM sleep regulation that produce dreams has highlighted the commonalities between the neural basis of REM sleep and emotional processing (overview Scarpelli et al., 2019). As shown in brain-imaging studies, there is augmented activity in brain regions such as the pontine tegmentum, thalamus and basal forebrain, the limbic and paralimbic structures during REM sleep, as compared to NREM sleep activities (e.g., Maquet et al., 2005) and waking states (e.g., Nofzinger et al., 1997). Other brain regions such as the dorsolateral PFC (dlPFC), precuneus, orbitofrontal cortex (OFC), and posterior cingulate gyrus are instead deactivated, compared to during waking states (e.g., Braun et al., 1997). These conditions may explain some of the transformed time perception, loss of executive functions, and the lack of insight that characterize dreams (Desseilles et al., 2011). Further, recurrent REM sleep features such as high vividness, bizarreness, and emotional load (e.g., Carr and Solomonova, 2018) that are salient during the dream experience also operate in cases of counterintuitive and supernatural agent dream cognition. This would be in line with the “continuity hypothesis” (e.g., Sterpenich et al., 2019), or with the suggestion that dreams and wakefulness share similar basic mechanisms. Consequently, during REM sleep and dreaming, most of the regions involved in emotional memory encoding are highly activated (e.g., Armony, 2013). This can be summarized as the emotional intensity of reported experience during REM sleep dreaming being explained by the higher activation of amygdaloid centers, the hippocampal formation, and the anterior cingulate cortex (ACC) during REM sleep states (e.g., Corsi-Cabrera et al., 2016).

Throughout the REM sleep period, several discrete physiological and neurological conditions occur. These include discharges in the autonomic nervous system, hormonal release, muscle paralysis and/or jolting, changes in blood pressure and heart rate, and high activation of the limbic region and the amygdala (detailed account in McNamara and Bulkeley, 2015; McNamara, 2016). In a summary by McNamara and Bulkeley (2015), during REM sleep, there is additional deactivation of the dorsolateral prefrontal cortex, the locus coeruleus, and the noradrenergic as well as the serotonergic systems, in addition to an activation of the dopaminergic and cholinergic circuits (also, e.g., Maquet et al., 1996; Hobson et al., 1998). The intricate co-occurrence of these brain activities during sleep states correlates with and may provide underpinnings for more higher level cognitive processes that generate supernatural agent concepts. There is a manifest difference, both in dream content and experience, between REM and NREM states, such that the former peak in negative emotions, nightmarish threat scenery, and bizarre imagery, while the latter manifest the opposite tendency (Hall and Van de Castle, 1966; Revonsuo, 2000a; Domhoff, 2003). It has been shown by McNamara et al. that scored aggression levels were lowest in NREM and wake reports, compared to REM sleep, and that imagery of friendliness signifies NREM states (McNamara et al., 2010). The correlation between apprehensive/non-apprehensive imagery and REM/NREM states respectively also suggests that the supernatural dream content is heavily affected. And indeed, various forms of aggressive, demonic, threatening, and predatory supernatural imagery prevail in REM states, while non-apprehensiveness, friendliness, and love characterize NREM imagery (Bulkeley, 2007; Nordin, 2011; McNamara and Bulkeley, 2015).

During dreams, the production of a self-model shifts or dissolves depending on neurological functioning, and a sense of involuntariness occurs, leading to the perception of other agents and entities as prime causal agents in, and of, the dream sequence. Consequently, a dream or sequence of dreams may be experienced as being caused by other agents, presumably supernatural ones, rather than the dreamer. These conditions manifest in alleged “religious delusions,” where supernatural agents are held to be the cause of the patient’s experience (McNamara and Bulkeley, 2015) such as manifested imagery of “demons” and evil spirits (McNamara and Bulkeley, 2015); indeed the lavishness of expression of religious delusions and abnormal experiences is more prevalent in patients with schizophrenia (e.g., Mohr et al., 2010) and sleep deprivation.

Overall, and as previously has been noted from cross-cultural accounts, supernatural agent dream cognition and imagery are common features of nightmares. Importantly, supernatural agent imagery is a common feature of extreme forms of terrifying nightmares manifested in sleep paralysis, incubus dreams, horrifying lucid dreams, and psychiatric disorders such as nocturnal panic attacks, all of which frequently arise during REM sleep (e.g., Nordin, 2011; McNamara et al., 2018). Even though these parasomnias may, like narcolepsy, be considered as sleep disorders in their own right, they are characteristically observed in a variety of psychiatric disorders, such as major depressive disorder, anxiety, post-traumatic stress disorder, attention-deficit/hyperactivity disorder, schizophrenia, bipolar disorder, borderline personality disorder, and other psychopathological conditions such as substance abuse (e.g., Kirov and Brand, 2014; de Sá and Mota-Rolim, 2016; Molendijk et al., 2017; McNamara et al., 2018; Tempesta et al., 2018; Baird et al., 2019).

Dreams are routinely forgotten, and only a few are preserved and conveyed to the waking state. On the other hand, certain types of dreams are memorable and are retained in memory and conscious awareness. Current cognitive research suggests that emotions influence many parts of cognition including memory (Slovic et al., 2007); hence emotions elicited in or by dreams are likely to influence whether and how dreams are remembered. Negative emotions are indeed common, and dream research suggests that 80% of dream reports refer to apprehensive experience (Hall and Van de Castle, 1966; Revonsuo, 2000a), of which fear is the most common, followed by anger and sadness. Other studies of dream reports demonstrate that 60% of *recurring* dreams and nightmares depict natural hazards or attacks by predatory or hostile beings (Robbins and Houshi, 1983). Being chased or attacked is also recurrent among those suffering from lifelong nightmares (Revonsuo, 2000a, p. 886). These recurring tendencies suggest that negative emotions such as anxiety, panic, and fear are important adaptive responses to situations in which reproductive fitness and survival are at stake (Marks and Nesse, 1994).

### Simulation and Prediction in Dreaming

From evolutionary and neurocognitive perspectives, a parsimonious suggestion is that dreaming operates as way of exercising simulation and testing predicted or hypothetical outcomes. This would basically entail a presupposition about modality cognition and an ability to represent prospective and alternate counterfactual states of affairs (e.g., Lewis, 1973). However, in counterintuitive religious concepts, counterfactuality is disputed, and the notions should not be conflated (e.g., Barrett, 2004a,b; c.f., Atran and Norenzayan, 2004), though both may belong to a broader category also including bizarreness, etc. The existence of counterfactual dream simulation of past and future states has been demonstrated (McNamara et al., 2002) and extends to the state of REM dreaming as *prospective coding* (Llewellyn, 2016) and encoding for episodic memories (Llewellyn, 2013). For example, dreaming seems to be involved in the generation and development of future goals, values/desires, and contents of daydreaming in relation to *episodic prospection* (Spuznar, 2010; McNamara and Bulkeley, 2015) of possible states of affairs and outcomes. A prominent approach for explaining such types of dream machinery is *threat simulation theory* (Revonsuo, 2000a; Valli and Revonsuo, 2007, 2009). In this view, some dreaming processes seen during REM and even perhaps in NREM sleep are evolved cognitive processes selected for in ancestral human environments and geared to simulating threatening events and rehearsing threat perception and threat avoidance skills, so as to enhance vigilance to threats in waking life. Nightmares about *survival threats*, aggression, misfortune, and accidents are ubiquitous. Themes of “being attacked” or threatened by enemies, wild animals, strangers, or monstrous entities prevail in the majority of men’s and women’s dreams (Hall and Van de Castle, 1966; Domhoff, 1996). Other adaptive simulation functions of dreaming have been suggested related to fitness pressures from costly social and sexual-selection signaling (McNamara, 2004), and simulation of social interaction (Brereton, 2000; c.f., Humphrey, 2000, pp. 953–953; Nielsen and Germain, 2000, pp. 978–979). Different versions of the “social simulation hypothesis”/“social mapping hypothesis” (Brereton, 2000) may be crucial in relation to social and sexual selection and partner choice. Furthermore, and more broadly, simulating scenarios about the intricacies of social life – intentions, interpersonal bonds, status competition, cooperation, alliances, trust, and so on – may improve social skills and have adaptive value (e.g., Franklin and Zyphur, 2005; c.f., Valli and Revonsuo, 2007, p. 113). Research on social perception and attribution of the theory of mind in dreams shows the significant amount of time dreamers spend pondering dream characters (Kahn and Hobson, 2005, pp. 48–57). Recent research indicates that REM sleep dreaming may serve other adaptive functions. For instance, neurobiological mechanisms and dreaming states during REM sleep may contradict the continuity hypothesis through incorporation, fragmentation, and reorganization of memories within dreams (Horton, 2017), a condition that suggests an enhancement of human heuristic inventiveness (Cai et al., 2009). Research also suggests that the occurrence of dreaming and REM sleep episodes operates as an adaptive interference, which integrates recurrent and recent memories into a broader vital context of the organism comprising emotions, basic needs, and individual genetic traits (Kirov, 2013). It has also recently been proposed that REM sleep dreaming could provide optimal conditions for the forming and updating of predictive coding, which will be further discussed in the next section (Hobson and Friston, 2012; Kirov, 2016).

### Dream Simulation, Prediction and Cognition of Agency

Threat simulation theory connects to other cognitive proclivities such as a “hypersensitive agency detection device,” HADD (Barrett, 2004a,b), and both may be instances of a general tendency of *precautionary psychology* (Boyer and Bergstrom, 2008). HADD refers to a composite cognitive mechanism responsible for the tendency to (over) attribute agency to phenomena in the environment, such as supernatural agents; the intuition of agency is rather spontaneous and automatic (Bargh, 1994), and activates seemingly innate systems for attributing and predicting intentions and motives of other agents, that is, the “theory of mind” or ToM (e.g., Leslie, et al., 2005). Theory of mind attribution has indeed been shown to be pervasive and dominant in dreams (Schweickert and Xi, 2010). Also, as aptly observed by McNamara and Bulkeley (2015), theory of mind attributions play a crucial role in modeling of supernatural agent cognition, for example as seen in the cognitive science of religion (e.g., Lawson, 2001; Barrett, 2004a,b, 2008). However, ToM attribution is not sufficient for providing a comprehensive characterization of religious dream imagery, and other dream properties may be crucial, such as a sense of peculiar value and reverence (McNamara and Bulkeley, 2015), or of strategically important information of cognitive relevance to the believer/dreamer (Boyer, 2001; Barrett, 2004a,b).

As indicated by the frequency of nightmares and apprehensive dreams, in REM sleep, the dreamers’ agency is highly impeded or absent. Experiences that involve a suspended acting self-model may account for an increased sense of the causal agentive role attributed to other special dream characters (McNamara and Bulkeley, 2015; McNamara, 2016). According to a predictive coding approach (e.g., Howhy, 2013; Clark, 2016), cognitive brain processing, to grossly simplify, strives to confirm its own prospective states, actions, and thought. Experiences of being in charge of one’s own actions, mental process, and sensory events derive from automatic and comparative processes between predicted and intended outcomes, such that if there is a match, the experience of self-causation increases, while a mismatch between prediction and actual outcomes encourages attribution of external causal agency (McNamara and Bulkeley, 2015). During REM dreaming, there is a downregulation of a self-model in relation to activation and intention, and hence a predictive gap. This may suggest that in dreaming, the mind simulates various counterfactual and possible scenarios, but the ascription of agency to ego-external agents results from unconscious responses to predictive failure and lack of sense of ego agency (McNamara and Bulkeley, 2015). These descriptions offer additional force to observations about people’s proneness to dream agency attribution, though it does not completely explain why *supernatural* agency would be the preferred imaginary construct in religious dream episodes. According to McNamara and Bulkeley, the prerequisite for the production of highly memorable SA dreams is the same as that for the generation of ordinary SA concepts or God concepts, and involves mental simulation of alternate beings/realities, ToM attribution, and reckoning of extreme or ultimate values (McNamara and Bulkeley, 2015). This would suggest that all humans are bestowed with a mind-brain system innately primed to regularly generate supernatural agent concepts in dreaming. However, such a proposal raises questions about why not everyone’s dreams contain SA images, whether non-believers’ dreams contain these images, and to what extent devotees of any religious system have dreams filled with SA imagery.

### Supernatural Agent Concepts and Minimal Counterintuitiveness Theory

The theory of counterintuitivity can partly be seen as an attempt to explain and model distinct recurrent features of supernatural or superhuman concepts. The core idea of minimal counterintuitiveness (MCI) theory is a notion of counterintuition that refers to violations, such as breaches and transfers, of intuitive expectations about basic ontological categories (e.g., Barrett, 2000, 2004a,b; Boyer, 1994, 2001; Pyysiäinen, 2001; and for a more comprehensive review of the literature, see, e.g., Barrett, 2008; Pyysiäinen, 2008; Purzycki and Willars, 2016). Such breaches and transfers hypothetically render (minimally) counterintuitive concepts *cognitively optimal* in cultural transmission and communication (Boyer and Ramble, 2001). MCI theory is concerned with why certain representations prevail and are catchier than others, and one prime cause of such catchiness seems to lie in how well they match our human conceptual systems rather than in the concepts themselves. There is little doubt that human minds are better equipped to processes certain types of information and phenomena, such as social information, over others. Such a proclivity of “maturational natural cognition” (McCauley, 2011) ought to impact how concepts and ideas are recalled and consequently communicated and transmitted. This points to the fact that MCI theory is grounded in an epidemiological account of culture (Sperber, 1996). Cognitive processing that construes counterintuitive outcomes suggests that humans entertain intuitions – implicit expectations regarding what kinds of things exist and their properties. These are called *intuitive ontologies* that are spontaneously applied in *category formation* and in our everyday interaction with and understanding of the environment (e.g., Boyer, 1994, 2001). In consequence, humans utilize intuitive expectations and construe distinctions between entities such as animate and inanimate objects, and between persons, animals, plants, artifacts, and natural or physical objects. These descriptions are very much in line with, and based upon, the well-known description of two systems of cognition: intuitive, fast online processes, and slow, reflective off-line processes (e.g., Barrett, 1999, 2008; McCauley, 2011; Kahneman, 2012), and research on domain-specific cognition and categorization (e.g., Keil, 1989, 1992; Rosengren et al., 1991; Leslie, 1995; Gellman, 1999; Inagaki and Hatano, 2002; Spelke and Kinzler, 2007). Counterintuitive notions such as invisible beings, animate mountains, living stones, and statues that can cry or fly, are catchy, seize people’s attention, and are memorable. By consequence, these items stand a greater chance of being communicated and selected over cognitively non-optimal concepts in cultural transmission. MCI theorists usually claim that cognitively optimal counterintuitive ideas form the backbone of significant traits in the cluster of religious phenomena and traditions, and explain the recurrence of certain types of concepts as resulting from cultural and cognitive selection. Still, MCI theory does not aim to account for religious concepts as making up some kind of naturally demarcated conceptual domain (e.g., Barrett, 2017). The original point was to account for why slightly counterintuitive concepts occur and endure between and within populations rather than maximally counterintuitive and perhaps many non-counterintuitive and bizarre concepts.

Numerous studies have tested and qualified the notion of counterintuitiveness by showing that modestly counterintuitive representations tend to be recalled and transmitted better than massively counterintuitive ones (Barrett and Nyhof, 2001; Boyer and Ramble, 2001); that once encoded, such MCI concepts are easier to retrieve than intuitive concepts; that the MCI effect seems to have a higher probability of impacting idea transmission among young adults and adolescents (Hornbeck and Barrett, 2013); and that contextual expectations and narrative embedding are crucial for the recall of modestly counterintuitive representations (Atran, 2002; Atran and Norenzayan, 2004; Gonce et al., 2006). This means that counterintuitive concepts are evaluated according to the contextual setting of which they are a part (Gonce et al., 2006). Other studies by Upal have suggested different types of minimally counterintuitive concepts based either on context-based models or on content (Upal, 2010). Research by Porubanova et al. suggests on the other hand that concepts that are culturally counter-schematic and unexpected are remembered better than concepts that violate ontological domain expectations (Porubanova et al., 2014). These studies show that notions of agents that breach both cultural-schema and domain-level expectations tend to be remembered better than concepts referring to object and artifacts (non-agents). The cultural transmission aspect of the MCI theory was also combined with other notions such as that of “inferential potential” and “strategic information” as an attractor and selection factor (Boyer, 2001, 2003).

The notion of “strategic information” suggests that any social information that would be relevant for and activate mental systems that regulate social interaction is strategic, under conditions where humans are incapable of having full access to all possible information of strategic bearing (Boyer, 2001, pp. 152–155). Strategic information need not refer to some ultimate importance or value, though the sense of urgency and importance probably derives from evolutionary and fitness-enhancing sensitivities (e.g., Nordin, 2015). Humans have imperfect access to all possible information of strategic relevance and consequently also to strategic information. People also seem to presume that others’ access to strategic information is incomplete or concealed, with the exception of SA agents, which are commonly attributed counterintuitive properties.

## Methodology

### Quantifying Counterintuitiveness in (Religious) Dream Reports

In order to measure and quantify counterintuitive properties in religious representation, Barrett constructed a more precise procedure for specification, “Barrett’s counterintuitiveness coding and quantifying scheme” – henceforth Barrett’s CI scheme (Barrett, 2008) – which will be applied in the present case to supernatural dreams. Barrett’s CI scheme enables predictions about the transmission advantage of score 1 counterintuitiveness. Any higher scoring counterintuitive breaches are likely to be re-represented in a simpler and less counterintuitive form. This may also suggest the occurrence of metonymic or metaphoric transfer of less counterintuitive representations. Coding and identifying counterintuitive concepts entails coding public representations (in our case speech acts of dream content) for their possible private representational structure (c.f., Sperber, 1996) and further, since it is likely that human minds generally strive for relevance and computational simplicity and efficiency (Sperber and Wilson, 1995), the coder should employ a *simplicity principle* regarding the way people conceptualize counterintuitive representations, and assume that the simplest and least counterintuitive concepts are employed (Barrett, 2008). The coding procedure involved the following six steps. First, we should identify the basic level category membership of the counterintuitive representation. Secondly, we should identify the ontological category(ies) of the allegedly counterintuitive representation, such as spatiality, physicality, biology, animacy, mentality (theory of mind), and universals. Thirdly, we should code the types of *transfers* of counterintuition as superscripts, with capitalized prefix; e.g., *Bhagawan* (Hindu God) manifesting as a gigantic stone pig speaking human language and offering advice and blessings is to be coded as “STATUE” (Biology + Mentality + Solid Object) = B(iology) + M(entality) STATUE. Fourthly, code any counterintuitive *breaches* of expectation as Superscript Lowercase Suffixes, e.g., *Invisible Mountain* (breaches of expectations of physicality). This item should be coded as Rock (or Mountain) = ROCK ^p(hysicality)^. If steps 3 and 4 (transfer and breaches) are combined, one may get such things as: *a growing and invisible statue* (transfer of expectation of Biology and breaches of expectations of Physicality), coded as Statue = ^B(iology)^ STATUE ^p(hysicality)^. There are further steps in Barrett’s CI scheme, for example coding complex breaches, such as breaches within breaches, using parentheses. The final step of the scheme quantifies counterintuitiveness by totaling the number of symbolic letters, and provides a tool for robust quantification and specification of counterintuitive modalities in dream narratives, while the previous steps in the scheme provide rigorous criteria for the coding.

### Research Questions

How common are counterintuitive properties in the context of allegedly religious dream contents? This article aims to (1) explore the topic of the pervasiveness of cognitive counterintuitiveness; (2) test Barrett’s counterintuitiveness coding and quantifying scheme (CI scheme) for counterintuitiveness in the context of religious dreaming by assessing intercoder reliability; and (3) to explore the actual occurrence, prevalence, and base rate frequency of counterintuitiveness in dream reports. In order to map and measure the counterintuitive content, Barrett’s CI scheme was employed and used according the following parameters: (1) degree of explication in terms of how manifest/implicit the counterintuitive content was in direct reports of dreams and in the dreamers’ pondering about the nature of SAs that appeared in the dreams; (2) the number of counterintuitive object in dream reports; (3) the magnitude and scores of counterintuitive transfer and breaches; (4) types of CI objects (animals, artifacts, humans, others) and frequency of agent-based CI in relation to object-based CI. Using such parameters enables a phenomenologically more elaborate and fine-grained, and hopefully non-trivial, measurement of the modalities and prevalence of counterintuitive content in religious dreaming. In this context, the obvious and principal traits of counterintuitive content will be traced in order to demonstrate their constitutive function in religious dreaming.

### Participants

The present study aimed to map the amount of counterintuitive content contained in purportedly strange dream reports and specifically religious dream imagery by means of interviews with mostly Hindu Nepalese informants in Nepal. Data were collected in central and western Nepal, in semi-urban and rural areas, but also around sacred sites such as temples (*mandir*), sites of pilgrimage (*tirtha*), and cremation precincts. A standard tactic for gaining the confidence and approval of prospective informants was to stay in a given public area for a while and unsystematically engage people in conversation leading to the question of whether they remembered any dream they had had. If so, prospective informants were asked whether they have had a strange dream or a dream where Bhagwan was seen. If the informant affirmed, this the interview would begin and last for approximately 1–2 h. Sixty participants were interviewed, of which 65% were male, and the mean age was 61 years (age range: 17–92 years).

### Interview Format

The structured to semi-structured interviews followed a questionnaire that dealt with topics such as whether the informants remember any special or strange dream; what happened in the dream; if the dream content was communicated to others or if religious experts were consulted; whether they have had an explicitly supernatural dream (about “Bhagwan,” “Devi,” demons, etc.); whether they appreciated counterintuitive more than bizarre content; how they trusted and valued the dream content and attributed truth to it; an emotional dream score derived by measuring fear, joy, happiness, and various other parameters; and a score assessing religiosity. Informants were requested to reiterate a dream that came to their mind, then briefly ponder on what they thought that the dream was about, and whether it required moral and behavioral changes. The informants were further asked to spontaneously reflect on such topics as the properties of the supernal agents that appeared in their dreams. About halfway through the interview, informants were asked whether they had strange dreams that contained any seemingly strange items such as a “whispering stone” or a “weeping statue.” The point was to cross-check whether the informants actually had manifest counterintuitive items in their narratives. As it happened, most informants had already mentioned some type of manifest counterintuitive items from their dream story, although some informants complemented their dream biography by confirming that they had had such strange dreams. This ensured that no obvious conformation effect, such as questions leading to statements from the informants that they otherwise would not have made, could be seen.

Most interviews were conducted by a field assistant and translator working with the Nepali- and Hindi-speaking informant. The standard English questionnaire needed translation into a Nepali version. The replies were retranslated into English for coding.

### The Hindu Nepalese Ethnography of Religious Dreaming

Before applying Barrett’s CI scheme, it is valuable to provide a realistic depiction about what is actually going on in these religious dreams and the content of the narrative context from which the dreams derive. A common way that informants indicated the presence of counterintuitive properties in their dream narratives was by means of phrases like: “*Shiva/Mahadev* appeared and advised me to conduct the puja”; “*Durga* came and made me wake up. Told me to take bath”; “I was sitting with *Bhagwan* somewhere in the mandir. Everyone was singing bhajan, and chanting mantra”; “*Bhagwan Pashupatinath* came in my dream and told me that the kind of life I was living must end.” However, such frequent expressions are merely indirect clues of counterintuitive content, and not its manifest expression. Below are two examples demonstrating how and to what extent manifest counterintuitive content occurred in the supernatural dream narratives.

The first example of a dream narrative includes one counterintuitive transfer of mentality to an artifact or object coded as ^**M**^**Artifact**, from the underscored section in the quote below:

“Bhagwan appeared as a half statue (Murti)/half man offering advice”; “Bhagwan appeared as a statue talking to me like a human”; “a big snake (Nag) appeared whispering”; “I was sleeping in heaven in an abnormal flying bed in the air”; “an unknown/unseen voice came into my dream and ordered me to stop drinking alcohol and eating meat (to become vegetarian)”; “a Shaligram (fossil) came into my dream. It told me that I should stop people from doing business with Shaligram. It is a pabitra (sacred) work”; “I was sleeping in heaven in an abnormal flying bed in the air. Many Bhagwans (gods) were there around me worshiping me and throwing flowers to me.”

The dream narrative in the second illustration comprises a number of counterintuitive properties such as breaches of universality in an artifact/object – **Bowl**^**u**^; transfer to a person of the physical property of causing combustion – ^**P**^**Agent**; and a breach where the dead come to life – ^**B**^**Corpse**, as demonstrated in the underscored part of the quote below:

“Among many dreams the dream that I like to talk most about is: Beggar wearing grey clothes who came to my house asking for rice/money. I went inside my house and took a bowl of rice from a basket and [poured it] … into his bag but the small bowl didn’t empty. The bag was full and the bowl was also empty. I got surprised [went]…inside again, opened the basket, but it was full [of] … *Shaligram* (sacred ammonite fossils, usually believed to relics of, and animated by, the deity *Vishnu Narayan*) and flowers. I could see *mandirs* and rivers inside the basket where *Bhagwans* were singing and dancing. I went and joined [the] *Bhagwans* and *Devetas* there. I was like a friend to them. They put me on a bamboo stretcher and threw [me] into the water. My legs and body [were tied] with a *Nag* (supernatural serpent). I could do nothing after that; I was put on a block of firewood. Some unknown people (actually could not remember what is was) produced fire from the mouth and set fire to the firewood. I was burnt and died. The fire went out and again I got off the block of firewood and came down, nothing has happened to my body, I was surprised in the dream too.”

## Results

In order to test and qualify Barrett’s CI scheme, intercoder reliability was tested and then the scheme was applied to measure and quantify some core modalities of counterintuition in dream narratives.

### Intercoder Reliability

**Figure 1:**
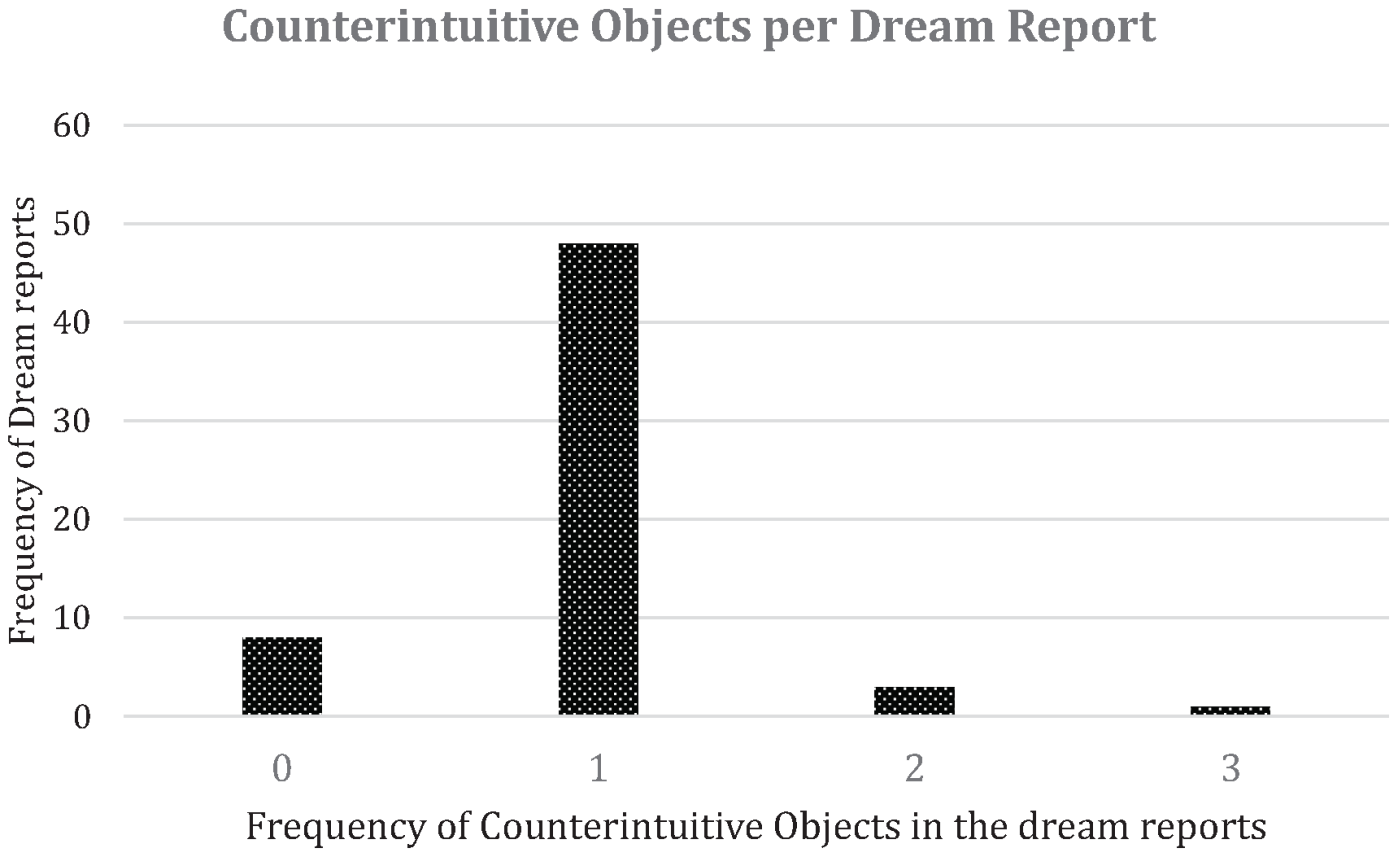
Visualization of the distribution of counterintuitive objects within dream reports, most dreams have counterintuitive objects, further one counterintuitive object per dream report is most common, only a minority of dream report have two or three counterintuitive object.

First, regarding the general occurrence of CI objects in dream narratives, the agreement between the two coders was 95%. To statistically test the consistency between coders, Kendall’s Tau-b was calculated. According to the statistical test, the intercoder reliability between the two coders was high and significant, τ = 0.875, *p* < 0.001 (*N* = 60 counterintuitive objects). The three dream reports with disagreement regarding CI objects were in two cases due to both coders missing 1 CI object each, as well as one misidentification of an object by one coder; when this was pointed out, both coders agreed. This analysis concluded that 52 of the 60 dream reports had at least 1 CI element, and that the total number of CI objects in all dream reports was 57 (see Figure 1). The 57 CI objects were independently coded into 21 different categories and the intercoder correlation between coders was high, with a Cohen’s Kappa of 0.88 (S.E. = 0.045, *p* < 0.001), which represent almost perfect agreement between the two coders. The dream reports that were in disagreement after the first coding were recoded into total agreement after a discussion. For example, in the case of a godly figure sitting on water, by appealing to norms about godly figures (they can sit on water) the coders decided that this object would be better coded as an Agent with a transfer of physicality than as Water with transfer of physicality. To further clarify, the disagreements were not so much about coding these figures as CI occurrences as about how to interpret a counterintuitive object in an ambiguous dream report; both coders agreed that if the dream report was interpreted in one way, the coding would have been in agreement. Overall, these results demonstrate the utility of the CI scheme for analyzing dream reports, but also that using cultural schemas to interpret CI occurrences reduces ambivalence in dream reports. Hence, knowledge about both Barrett’s CI scheme and normative beliefs about supernatural agents will improve intercoder reliability when coding dream reports.

### Counterintuitive Objects per Dream Report

To investigate the frequency of counterintuitive objects per dream (Aim 1), we coded the number of CI objects that were reported per dream. Fifty-two of the 60 dream reports had at least one counterintuitive object (87%), suggesting that counterintuitive objects are relatively common in dreams, or alternatively that people remember or wish to communicate dreams with counterintuitive objects more than dreams without counterintuitive objects, which also would lead to a high frequency of dreams with counterintuitive objects being reported. Furthermore, in line with the theoretical suggestion that counterintuitive objects are related to cognitive load, 48 dream reports had one counterintuitive object, while only three dream reports had two counterintuitive objects, and one single dream report had three counterintuitive objects (see Figure 1). So, while counterintuitive objects occur in the majority of dream reports, making them frequent across dreams, they are relatively infrequent within dreams, with an absolute minority of dream reports having more than one counterintuitive object.

### Counterintuitiveness Scores of Objects

Each counterintuitive object can theoretically have several breaches and transfers, or a combination of both. This is quantified by adopting the score of the sixth step in Barrett’s CI scheme, where each breach or transfer gives the object an additional point. However, previous research on folktales suggests that most counterintuitive objects have a score of 1 (meaning they only have one breach or transfer) and that few counterintuitive objects have a higher score than 3. In line with these findings, the most common score in dream reports was 1. Specifically, 54 objects had a counterintuitive score of 1, three had a counterintuitive score of 2. There were no objects with a score higher than 2.

### Most Frequent Types of Counterintuitive Objects

**Figure 2:**
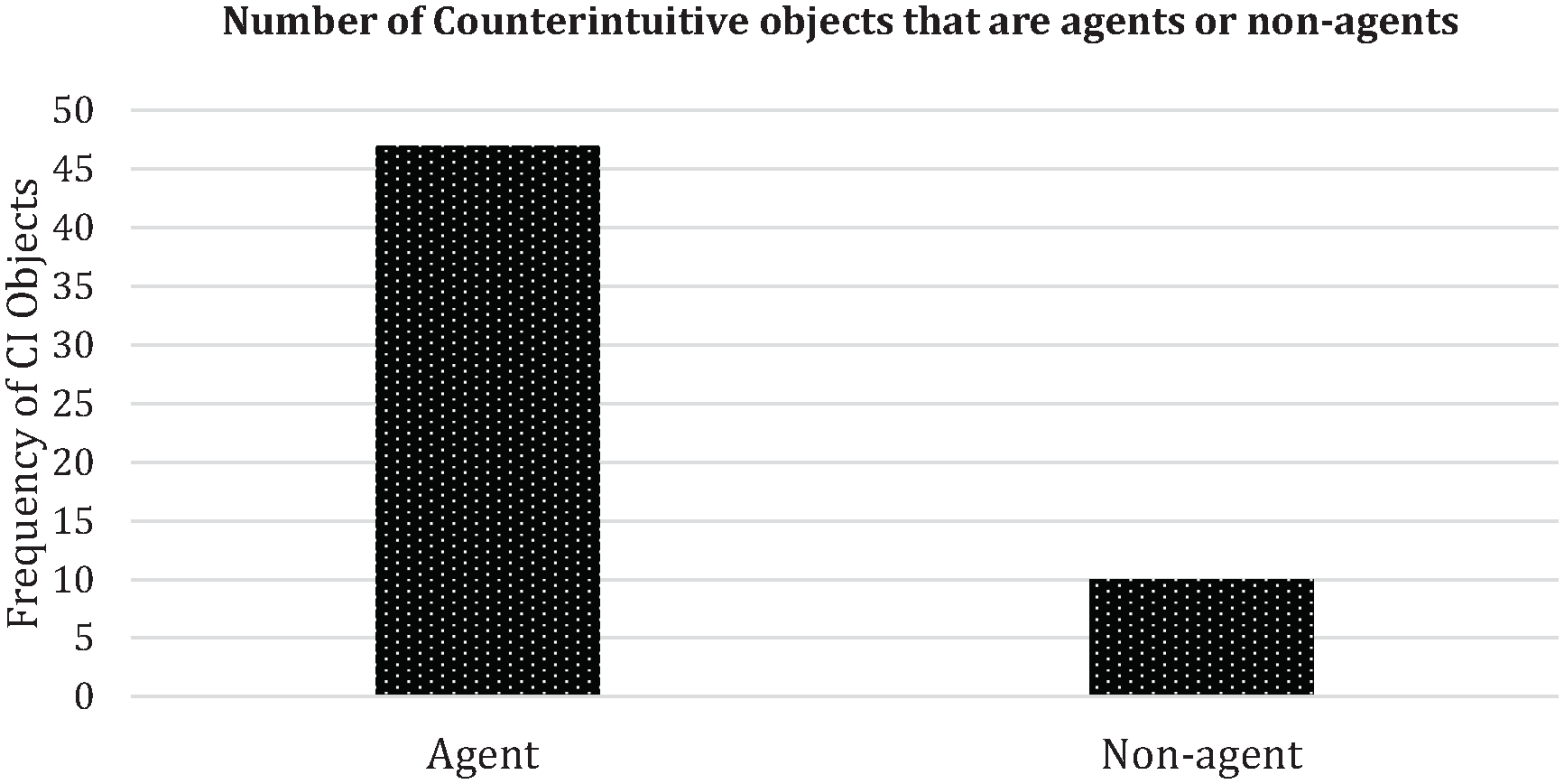
The frequency of counterintuitive objects categorized into agents vs. non-agent, as seen in figure the majority of counterintuitive objects are agents.

**Figure 3:**
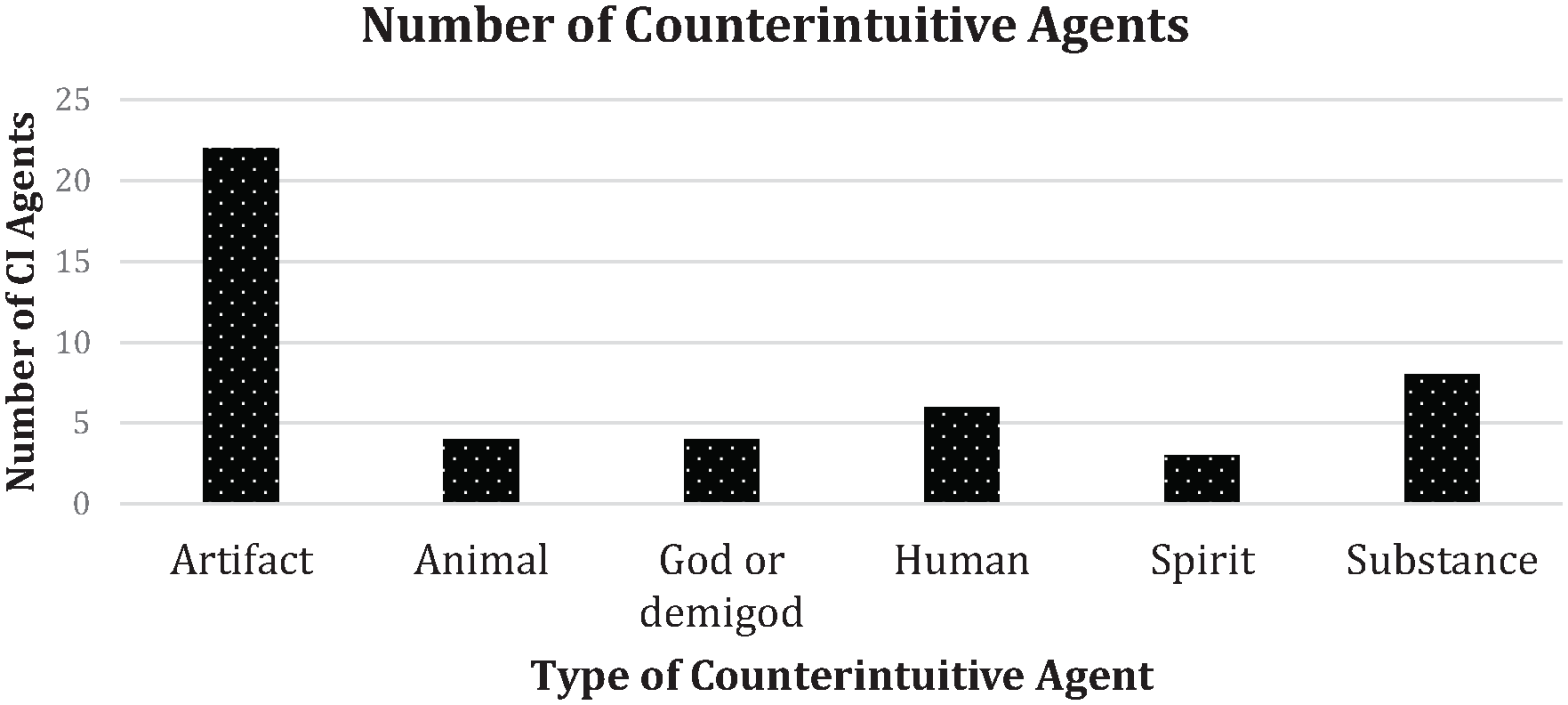
The frequency of different types of agents in the dream reports, most of the agents in the dream report are artifacts.

Most counterintuitive objects (47 out of 57) were agents, that is, objects that activated either mentality or animacy expectations or artifacts, humans, animals, substances, spirits, demigods, or gods with counterintuitive properties that acted intentionally in goal-directed ways. The most common agents in the dream reports were artifacts, often statues of gods that activated either mentality or animacy expectations (see Figures 2, 3). However, the second most common type of agents were bodiless voices (substance) that activated mentality expectations. Apart from these forms of counterintuitive objects with agent properties, a few cases (10 out of 57) consisted of counterintuitive objects with non-agent properties, such as impossible artifacts (e.g., a container that is larger on the inside than on the outside), luminous objects, etc. The prevalence of counterintuitive objects with agent properties may, at least according to the proposed byproduct and cognitive-relevance approach employed in this analysis, be explained according the principle that they entertain more cognitive routines and heuristics specialized on social information, interaction, inference, and utility in the least processing-costly way. This suggests that such proclivities predominate in the dreaming processes relating to imagery production, retrieval, recall, and communicative transmission. This supports Boyer’s statement (Boyer, 2001) and Barrett et al. (2009) finding that in particular, counterintuitive intentional agent concepts obtain cultural transmission advantages over other sorts of counterintuitive concepts. The idea that intentional agent concepts should have transmission advantages derives from the theory of relevance (e.g., Wilson & Sperber, 1996), on which basis it is suggested that agent concepts have a more extensive inferential potential than other concepts, since they rely on a pervasive set of systems for social and moral interaction, and in the case of SA concepts, on strategic knowledge, etc.

## Discussion and Limitations

Do so-called “religious dreaming” and SA imagery overproduce counterintuitive (below) imagery? Or do cultural and religious schemata provide an evaluative and conceptual context that enhances memorability and attention, and hence the selection and attraction of counterintuitive SA concepts? Although agent concepts predominate in dreams with counterintuitive contents, there are also cases of non-agentive counterintuitive dreams (below, empirical section).

Also, why should counterintuitive SA dreaming be limited to religious correlates? One common and general suggestion is that counterintuition, besides bringing strong and vivid emotions, improves the memorability, salience, and inferential potential of religious dreams, rendering these representations successful in cultural transmission and selection. Furthermore, the fact that certain cultural environments ascribe higher value to supernaturalist imagination and representations would indicate the operation of schemata that in themselves would support the memorability and transmission advantage of religious dreams.

The aim of the article was to map and align contemporary theorizing regarding CI schemes with empirical qualification of the prosaic hypothesis about the predominance of supernaturalism in allegedly religious dreaming. Our results suggest that exploring the pervasiveness of cognitive counterintuitiveness in dream reports is a promising way of merging theories about religious dreaming with MCI theories. They further suggest that Barrett’s CI scheme is useful for coding counterintuitiveness in the context of religious dreaming. Lastly, we confirm that counterintuitiveness is extremely prevalent in dream reports. To our knowledge, this article is the first to apply Barrett’s CI scheme to dream reports, and hence this undertaking contributes to the methodology and understanding of religious dream cognition as well as establishing cross-cultural base rates of counterintuitiveness in dreams for future research.

These conditions may be summarized according to a common model in the cognitive science of religion: just as the predisposition to detect and infer agency increases in situations of salient experience, urgency, and uncertainty (Barrett, 2004a, pp. 39–40), noteworthy dreams and nightmares with strong negative emotions and existential worry presuppose such a sense of urgency. Accordingly, stress from apprehensive experiences in dreams contributes to supernatural beliefs because: (1) supernatural agent concepts are particularly relevant, salient, and inferentially rich (strategic knowledge and morality); (2) they are usually already part of the believer’s explanatory repertoire; and (3) they are culturally institutionalized as “special” cues that need interpretative decoding by experts and/or with manuals. In dreams, the supernatural agent concepts are particularly relevant and attractive to draw upon, and they outcompete other explanations. Accordingly, intentional actions by SAs with counterintuitive properties are likely to be held to be the cause or content of the dream. This would, according to HADD, be because salient dreams are conceived as traces of, or communicative signs from, other agents (Barrett, 2004a, pp. 36–37). However, previous accounts seem unable to explain why seemingly religious dreaming would (1) contain *manifest* supernatural agent imagery and (2) *manifest* counterintuitive properties of SA dream imagery in the first place.

Has the present study overestimated the amount of counterintuitive content in the dream reports? Major factors that could have led to overestimation, exaggeration, or distortion could be effects of various biases on the selection of informants, the interviews, the coding procedure, or the compilation and amalgamation of interview data. For example, Barrett et al. (2009) highlight that the distillation process in the collecting of folktales may overestimate the frequency of counterintuitiveness by combining oral versions into a written version, where such a compiled version would not operate under the same mnemonic limitations as verbal communication of narratives and stories. The present research tackles some of the methodological and conceptual challenges, such as issues about biased coding and data compilation. It demonstrates a slight discrepancy between the field assistant’s and supervisor’s identifications of allegedly counterintuitive traits in dream reports; however high agreement between the coders was registered, as previously demonstrated. General corroboration of the applicability of the CI scheme was given in Barrett et al. (2009), supporting the use of the strategy as common approach for identifying and quantifying counterintuitiveness. Another relevant and related topic that this article has not addressed is whether there is a cognitively optimum number of counterintuitive items in a reported dream narrative. Although the data point to instances where different counterintuitive items occur in a reported dream narrative, usually there is one counterintuitive item per dream narrative. This may agree with what Norenzayan et al. (2006) suggest as a base rate optimum for cultural transmission of sets of narratives as a whole. For example, it is likely that the transmission of oral material such as folktales and narratives selects for MCIs, while the cultural and institutional support for these items (Barrett et al., 2009) may increase the prevalence, magnitude, and extension of counterintuitive properties. The topic of a cultural transmission advantage or optimal number of counterintuitive representations in reported dream narratives may seem irrelevant for the actual occurrence of CI notions in dreams. However, there certainly is an intricate connection between the counterintuitive representations in a given cultural environment, their institutional and sacred value, asserted “specialness,” religious “affordance” (c.f., Gibson, 1977), credibility enhancing potential (Henrich, 2009; Turpin et al., 2018), and the overt communicative exposure and priming effect they exert. Dreams are affected by and “situated” in social experiences, schemata, artifacts, and iconography developed and shaped in local cultural environments. The fact that counterintuitive contents in general, and counterintuitive intentional agent concepts in particular, are so predominant in the dream material from the interviews strongly suggests that this type of processing and information has a transmission advantage. It also offers some general support for cultural transmission models based on selection and attractor factors of evolved cognition and intuitions (e.g., Sperber and Hirschfeld, 2004; Claidiére and Sperber, 2007; Morin, 2016), even if these approaches are broad in scope and not aimed at explaining why CI imagery occurs to begin with.

Most dreams are routinely forgotten and only a few are selectively conveyed to the waking state. Some are sufficiently memorable to be retained in an individual’s memory and conscious awareness. Religious or supernatural dream cognition seems to be highly effective in generating memorable experiences, because it includes supernatural agent concepts that are counterintuitive, and thus highly likely to be preserved in memory and further culturally transmitted (Nordin, 2011). This would explain the high frequency of CI in the dream reports. Consequently, the theory of counterintuitive processing – in combination with a readiness to attend to strategic information, cognition of contagion and essentialist reasoning related to the magical and ritualistic use of SA agents, and their cultural schematization and institutionalization – perhaps contributes to a fuller picture of the dream and culture interface.

As a final note, because dreams and dreaming are understood differently within different research fields (neurology, psychology, and anthropology), different research methods will be needed to capture dreams and dreaming. What constitutes a “true sample” of dreams might also differ between research fields. The theories on which we base our research state that dream research respondents are more likely to remember, and also transmit, dreams that contain CI. In light of this, our sample will be biased toward dreams with CI content. From one perspective, this is a limitation; however, it is also exactly what we expected and serves as confirmation of our hypothesis and the theories that it rests upon. However, to remedy this respondent bias, it is advisable to try to assess dreams that normally would not be communicated. There are reasons to believe that respondents do not generally report mundane or boring dreams, and because memory is a living process, these dreams will be forgotten. Instructing respondents to write down every dream they have during a week, even the most mundane dreams, should, in accordance with our thinking, produce a lower rate of CI in those dream reports. However, this might not fully correct the respondent bias, because memories of dreams are very fleeting, and dreams can be forgotten in the very instant when they are remembered. Further, an assessment like this would be different from what we tried to assess in our study. Our goal was to assess dreams that are remembered automatically and communicated freely, as these are the kinds of dreams that best represent what constitutes dreams within a culture.

## Conclusion

The MCI model has been one viable area of research and debate in the cognitive and evolutionary study of religion. An issue that these scholarly debates and enquiries highlight is to what extent counterintuitiveness is a robust phenomenon and a recurrent feature of significance in the culturally variable repertory of supernatural agent beliefs. Other issues concern topics such as seemingly “non-religious” counterintuition, memory, the interface with bizarreness and counterintuitiveness, cultural schemas, and other transmission biases. Counterintuitiveness has been analyzed and confirmed in different cultural and religious contexts, though not systematically in the context of religious dream content. On the other hand, as has been demonstrated in this article, supernaturalism in dreaming has indeed been reported from many contexts and sources. The present article has offered support for the applicability of Barrett’s CI scheme and MCI theory in explaining the crucial traits of SA and religious dreaming by measuring pivotal quantities and modalities of counterintuitive content. Employing a standard assessment of counterintuitiveness minimizes inter-study variability while improving comparability and base rates, and advancing the empirical exploration of MCI theory and comparable theories related to SA and counterintuitive concepts. Importantly, this article has demonstrated the prevalence of counterintuition in (religious) dreaming by quantifying a selected array of modalities that serve as markers of counterintuitive processing by applying Barrett’s CI scheme and testing its methodological validity and efficacy.

## Data Availability

All datasets generated for this study are included in the manuscript and/or the supplementary files.

## Ethics Statement

- This research fully complies with the ethical guidelines of the Swedish Research Council, including with regard to informed consent, strategies of anonymization, and informing the interviewees about the consequences of their participation. All reasonable efforts were made to ensure that the ethnographic interviews and research process would not jeopardize participants’ integrity. By following these conditions, the study avoided potential ethical problems from the start.
- Before each ethnographic interview, potential participants were informed about the consequences of their participation and told that they were contributing to scientific research on dreaming and the Hindu religion. It was made clear to them that they were free (1) to choose whether to take part in the interview, (2) to decline to answer any question, and (3) to stop the interview at any stage. Furthermore, they were fully informed that personal and sensitive topics were not part of the questionnaire and were not of interest for the research project. Interviewees gave their verbal informed consent, and strategies for anonymization were used.
- No vulnerable populations were involved. Regarding our choice of verbal informed consent, the use of written informed consent from informants is not practiced (in the social sciences) when ethical concerns are estimated to be largely absent and the procedures adhere to the guidelines of the Swedish Research Council. Regarding ethical approval, when research projects include topics of sensitive ethical nature, they must be approved by a committee at the local university in Sweden; however, the present project never touched on or dealt with such questions.
- As a consequence of the above-mentioned facts, ethical approval was not required as per the applicable institutional and national guidelines and regulations in Sweden.
- Finally, the present study was conducted in the developing country of Nepal, where seeking written informed consent would have been difficult, impractical, and even potentially counterproductive from an ethical perspective. This is because many persons in this sociocultural context may believe that signing formal (and foreign) documents could lead to legal problems and trouble with the authorities.

## Author Contributions

AN is the main author that with the aid of a field assistant collected data in Nepal. PB is co-author and worked with data collection strategy and data analysis.

### Conflict of Interest Statement

The authors declare that the research was conducted in the absence of any commercial or financial relationships that could be construed as a potential conflict of interest.
